# Helping Frontline Workers in Texas—A Framework for Resource Development

**DOI:** 10.3390/ijerph20206935

**Published:** 2023-10-17

**Authors:** Karima Lalani, Meredith O’Neal, Simone Lee Joannou, Bhanumathi Gopal, Tiffany Champagne-Langabeer

**Affiliations:** 1 School of Public Health, University of Washington, Seattle, WA 98195, USA; klalani@uw.edu; 2 McWilliams School of Biomedical Informatics, The University of Texas Health Science Center at Houston (UTHealth Houston), Houston, TX 77030, USA

**Keywords:** mental health, substance use disorder, first responders, interprofessional education, COVID-19

## Abstract

First responders disproportionately experience occupational stress when compared to the general population, and COVID-19 has exacerbated this stress. The nature of their duties as law enforcement officers, firefighters, and medics exposes them to repeated trauma, increasing their risk of developing a broad array of mental health issues, including post-traumatic stress disorder (PTSD), substance use disorder (SUD), and compassion fatigue. This paper describes the need for resources for frontline workers and provides a framework for creating and implementing resources. A team of interdisciplinary subject matter experts developed two major resources. The first resource was a 24/7 helpline to support first responders and healthcare workers experiencing substance use or mental health concerns. The second resource was the First Responders Educational Campaign, which developed and delivered focused training modules on useful topics covering substance use and mental health concerns as they pertain to this workforce. Utilizing core interprofessional principles, content was sourced from multiple disciplines and contrasting perspectives to provide a comprehensive understanding of mental health and substance use issues. The curriculum was designed so that the content was interdisciplinary, interprofessional, and accessible to audiences across disciplines and professions. After engaging more than 1500 individuals, resources developed here have augmented mental health and substance use support resources available to the target population.

## 1. Introduction

First responders disproportionately experience occupational stress when compared to the general population. The nature of their duties as law enforcement officers, firefighters, and medics exposes them to repeated trauma, often resulting in the development of post-traumatic stress disorder (PTSD), substance use disorder (SUD), and compassion fatigue [1,2,3]. A documented occupational risk factor for Emergency Medical Services (EMS) workers is high rates of alcohol and drug use [3]. At its most extreme, the combined effects of alcohol use, PTSD, stress, and lack of sleep commonly felt by firefighters has been associated with suicidal ideation [4]. In one of the few published studies, researchers report that female first responders had rates of substance use that are 3× that of the general population [5]. Yet, EMS workers are often reluctant to seek care to avoid disciplinary action, stigma, or other unspoken “codes of silence” that are prevalent among first responders [6]. Mental health disorders also frequently co-occur in the EMS workforce, and the rate of death by suicide is well over 2× that of the general population [7,8].

Healthcare workers (HCWs) similarly experience occupational stress, particularly since the COVID-19 pandemic [9,10,11,12]. Research shows that HCWs increasingly employ avoidant coping strategies post-pandemic and report higher levels of alcohol use [11]. Similarly, rates of opioid-related deaths increased during 2020 and 2021 [13]. Self-paced, educational modules may offer support related to resilience, self-efficacy, and mindfulness—skills frequently needed during times of high stress [14]. Continuing education modules that provide both theoretical and practical knowledge while allowing learners to apply their understanding of stress management techniques have proven to be the most efficacious in settings where the patients were HCWs [15]. The goal of this project was to create an extensive system of care for first responders to address the need for counseling for substance use and anxiety disorder and mental health needs.

## 2. Materials and Methods

The resource framework was developed by a group of interdisciplinary researchers at the Center for Health Systems Analytics (CHSA) within the McWilliams School of Biomedical Informatics at the University of Texas Health Science Center at Houston (UTHealth Houston). CHSA collaborated with community stakeholders, including the Texas Office of Emergency Medical Services (EMS) and the Texas Targeted Opioid Response (TTOR) to develop resources for frontline workers. The first was an educational campaign focused on creating content to address a gap in knowledge and widespread stigma and misinformation about mental health and substance use. The second resource was a 24/7, anonymous telephonic helpline for frontline workers experiencing substance use or mental health concerns.

These objectives combined to advance the overarching goal of supporting first responders and healthcare workers in acknowledgement of the unique risk factors faced by the professionals [16]. The following sections outline and discuss programmatic implementation strategies and operational protocol to provide a critical look and how-to guide for creating valuable resources for the target population, including best practices, challenges, and adaptations.

### 2.1. Frontline Worker Educational Campaign

The first resource created was the Frontline Worker Educational Campaign (FWEC). FWEC is a free, research-based learning service implemented to provide first responders and HCWs across the state with information regarding substance use and mental health disorders. At inception, the primary audience was first responders. However, the project later expanded to include healthcare professionals. Educational materials covered mental health concerns such as depression, anxiety, PTSD, compassion fatigue, and substance use, including alcohol and opioid use disorders. CHSA created asynchronous online training modules that provided frontline workers with the tools and information necessary to recognize and address symptoms of substance use and mental health disorders in themselves or others. Synchronous training and advocacy options, both in-person and virtual, were also offered to group learning sessions for departments seeking real-time presentations and live, question-and-answer sessions. Owing to COVID-19 restrictions, synchronous trainings were predominantly conducted virtually via webinar. Curriculum development started in late 2019 with a go-live date of 1 April 2020.

All educational materials incorporated evidence-based, practical applications. CHSA researchers were well-versed in many academic disciplines that contributed to the curriculum design that facilitated the objectives of the project. This involved incorporating the subject matter expertise of a medical anthropologist, a mental health ethicist, a public health expert, and an experimental psychologist. Team members provided other essential insights with backgrounds in counseling, behavioral economics, criminal justice, medicine, nursing, peer recovery, data science, emergency medicine, nutritional science, and instructional design, among others. Given that the target audience received information from perspectives beyond their formal education, the authors found that this strategy satisfied the essence of interprofessional education and students learned from each other.

Asynchronous training modules were effectively delivered through Learning Management Systems designed for continuing education. The selection of the preferred Learning Management System (LMS) was dependent on the resources and needs of the training development team. The team was furnished with access to Canvas, the LMS platform utilized at the university, and all courses were designed with accessibility principles using a distance education specialist.

To begin, a wireframe outline was developed. The authors determined the core content objectives and introduced and established the problem in current research-based evidence. Next, the relevant problems faced by the first responder and HCWs were identified. It was then enumerated how these issues affected individuals in the workforce and their peers along with actionable tools to address each concern.

The content for all trainings, synchronous and asynchronous, was designed as a lecture presentation, utilizing online presentation software. Basic presentation design principles were followed, i.e., using minimal text per slide, utilizing graphics and animations, recording transcripts, embedding video or website links, and staging multiple review opportunities. The design was simplified to deliver content as economically and effectively as possible while sustaining the learners’ attention. Content was delivered in a manner suitable for persons of all learning styles, including visual and auditory by providing transcripts. In addition, all content was designed to ensure optimum accessibility to support learners with disabilities. For asynchronous modules, this included a narrated video format of the presentation and a slide deck with additional notes for review.

All synchronous modules, delivered either virtually or in-person, were conducted as lectures or workshops. The curricula of these modules rarely differed from the asynchronous options, although they may have been slightly tailored to specific audiences.

Demographic data were captured, and quizzes to assess comprehension were administered during asynchronous modules. An optional satisfaction survey was also provided during asynchronous modules. Data collection differed in synchronous sessions, where face-to-face contact was most prevalent. The trainer found that survey collection and quiz administration distracted from the flow of the session or was otherwise discouraged, and it was quickly abandoned. The authors plan to resume data collection under a shared agreement in the future.

### 2.2. Heroes Helpline

The second resource was a helpline to support first responders and HWCs experiencing substance use or mental health concerns. The idea for a helpline for first responders originated in 2017 from the Texas Office of EMS, which recognized the need for such a resource. In 2018, the Texas Office of EMS and the Texas Targeted Opioid Response team began searching for funding opportunities. Once secured, these stakeholders approached UTHealth Houston’s Center for Health Systems Analytics in 2019 to operationalize the idea. By January 2020, CHSA set a launch date for 1 April 2020. Despite the onset of the COVID-19 pandemic, targets were met and the helpline launched on 1 April 2020. The following paragraphs detail the procedures used to create what is now the Heroes Helpline.

The development process began when the decision to create a helpline was made and approved. The first phase was the conception phase and included the planning for the helpline. The second phase was the implementation phase and discussed the conversion of the helpline from launch stage to becoming operational. The third phase was the active phase and included ongoing efforts to keep the helpline in service and to promote educational content throughout the process.

Developing a helpline required significant planning and infrastructure. The conception phase entailed key considerations, including staffing, availability, and confidentiality. Based on the research interests and geographical location of the Center for Health Systems Analytics, the target population was determined to be first responders in Texas only. The team decided to offer a 24/7, confidential, telephonic helpline as a secure resource with flexible hours. Peer recovery support specialists were enlisted to answer calls and determined that the helpline should be available to callers at all hours of the day on every day of the year. Peer recovery support specialists offered a non-clinical, lived experience for people experiencing substance use and mental health concerns, and this unique perspective provided an accepting space for frontline workers to discuss their concerns and experiences openly. Peer recovery coaches were certified by the state, trained internally for a minimum of 6 months, and were supervised by a doctoral-level counselor. A protocol was developed for immediate action, which entailed calling 911 to the caller’s address.

Next, the implementation phase began, starting with a list of actionable items. The lead project coordinator worked with other team members to obtain a toll-free number, obtain Institutional Review Board approval, hire peer specialists, develop an answering protocol and script, and build a database for electronic record management.

The study team employed REDCap, a secure web platform, for research data management. Using REDCap capabilities, several surveys were created and tailored to data collection needs. The first was an enrollment survey to collect demographic and contact information from callers. Second, an informed consent form was employed to document the collection of verbal consent for every caller and written consent, where applicable. Referral and follow-up surveys were developed to document referrals and follow-up outcomes as necessary. Data were collected monthly over a three-year period from 1 April 2020, until 30 April 2023.

Helpline staff established relationships with treatment providers across the state to act as a referral network for callers. To assist in this effort, a resource network document was created, detailing services and contact information for these providers. Complementary providers included the Trauma and Resilience Center at UT Houston’s Psychiatry department. This program specializes in the mental health of veterans and first responders. Complementary providers typically included a multidisciplinary team to provide treatment, research, and education devoted to helping people experiencing psychological problems after traumatic life experiences, focusing on veterans and first responders. The project coordinator also contracted a healthcare answering service to manage holidays and after-hours calls. Through the established network, an answering service provider specializing in healthcare communications was enlisted to briefly screen callers before transcribing a message or patching the caller to on-call helpline staff.

The helpline became operational on 1 April 2020. Once operational, protocols were re-assessed and adjusted as needed. After receiving several calls, it was determined that the natural flow of conversation differed from the original script. As a result, the script was reworked to reflect practical application. This adaptation improved caller satisfaction and call handling.

Hypothesizing that first responders might be reluctant to place a phone call to the helpline, the team researched and ultimately established a HIPAA-compliant online chat option. Using a pre-approved vendor, this option became available in late 2021.

## 3. Results

Table 1 provides descriptive statistics about the educational campaign participants as of 30 April 2023. There were a total of 2151 participants in the trainings. The majority 68.9% (*N* = 1482) were non-Hispanic/Latino, although 7.7% (*N* = 165) declined to respond to the question. Nearly 20% (*N* = 428) declined to respond to the question regarding race. Those who answered were Asian 25.4% (*N* = 546), Black/African American 23.8% (*N* = 511), and White 22.9% (*N* = 493). Males were a slight majority at 46.9% (*N* = 1009).

**Figure 1:**
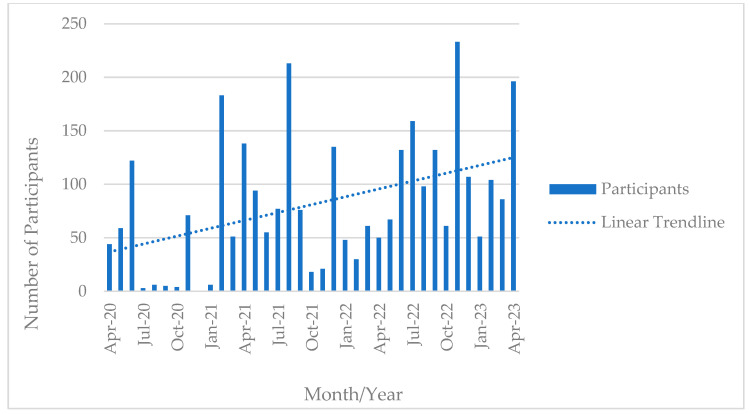
Educational campaign for frontline workers, participants by Month, 1 April 2020–30 April 2023.

Figure 1 displays the number of participants per month. The first year (2020) was low in participant numbers as the team developed relationships and expanded the educational offerings. Additionally, this was a time of labor shortages and limited time was available for training the first responders. The assumptions are reflected by the low number of participants. Certain months allowed for very large training opportunities (e.g., February 2021, August 2021, July 2022, November 2022, and April 2023). These included trainings at local and national conferences where first responders were offered free continuing education opportunities.

The center started with a short list of topics of greatest need and of interest, namely substance use disorder specific to first responders and techniques for coping with anxiety. Using subject matter experts, the material was expanded to include evidence-based practices, which include emotional freedom technique (EFT) or more commonly known as tapping, meditation, deep breathing, and mindfulness [17]. These skills have been embedded into various modules throughout the curriculum. Based on data available through the end of 30 April 2023, Table 2 shows the course offerings by topic. This provides a complete list of the variety of topics created by the center for educating the frontline workforce on substance use, suicide, alcohol use, and stress-relieving techniques for anxiety disorders. Modules are updated on an ongoing basis and new modules are added every two to three months.

**Figure 2:**
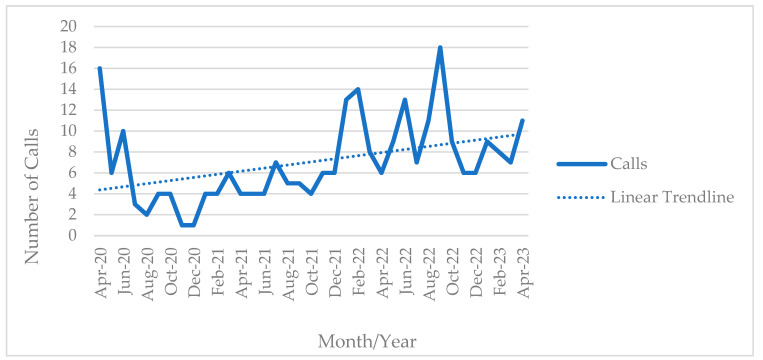
Calls to the Heroes Helpline since inception, April 2020–April 2023, *N* = 261.

Call volumes are shown in Figure 2. This highlights the total number of calls to the helpline calls by month. April 2020 opened to 16 calls, the second highest only after September 2022 (*N* = 18). December 2020 was the lowest month on record where only one caller was recorded. Other months averaged between four and eight calls per the linear trend line. Calls came from emergency medical services employees, firefighters, law enforcement officers, and healthcare workers. Some callers were categorized as ‘informational callers’, usually from other treatment providers or first responder agencies. Satisfaction surveys were optional for willing callers. Results from satisfaction surveys were used to refine the services offered and enhance community relationships to provide additional services to frontline workers in crisis.

## 4. Discussion

The goal of this program was to fill a gap in the healthcare system for confidential education and counseling services for first responders in Texas suffering from substance use disorder, anxiety or PTSD, and mental health challenges. A large educational program was developed and delivered both in-person and online; the online program was both synchronous and asynchronous. The educational modules received a substantial response, as evidenced by enrollment data. During the same time, a 24 h helpline was launched and coincided with the COVID-19 pandemic.

Due to the limitations posed by the COVID-19 social distancing measures, curriculum accommodations were made based on considerations of social and experiential learning. This was performed by incorporating exercises that allowed for decentering, and content that encouraged empathy, moral imagination, and reflection [18]. These exercises may have been more effective in a live group training session; however, our research is supported by other programs initiated during this time with positive results. One web-based program, which provided mind–body resilience training, demonstrated feasibility with a group of 42 first responders, resulting in greater emotional regulation and stress tolerance [19].

The curriculum design employed materials that served individuals within any categorized learning style [20]. For example, the utilization of a narrated video met the condition of active experimentation, as did the implementation of staggered review quizzes. Within the audio–visual modality, the trainings provided supplementary, text-based content for those learners who favored abstract conceptualization. Using recent data supporting the content and first-hand personal accounts allowed learners to relate and engage in reflective observation. Guided skills videos enabled individuals to learn via active experimentation, as illustrated in the breathing and progressive muscle relaxation techniques demonstrated in the Understanding and Coping with Anxiety module.

As for the more abstract dimensions of learning, the trainings aimed to furnish learners with information that allowed them to answer questions about who they are personally and professionally; how they acquired the knowledge they did, why expanding that knowledge is valuable, what it might take for them to grow said knowledge bases, and whether their professional knowledge can or should include additional information. In the context of these training modules, this was essential for addressing beliefs that otherwise inhibit the capability and willingness of individuals to seek appropriate support for themselves or, indeed, to provide it for their peers and colleagues.

Upon resuming continuing in-person education, training content was enriched to include experiential and social learning principles, largely based upon existing frameworks established in disaster training scenarios [21]. For instance, the curriculum was augmented to ensure that learners actively participate in their learning and have opportunities for the inquiry process in real-time and access to a more intimate exchange of perspectives. Interpersonal communication exercises were also more effective in live classroom settings. Non-verbal communication was essential to identifying and addressing mental health and substance use concerns. Witnessing various non-verbal communication modes delivered by different professionals served to bolster the skills that learners were developing.

Although the anonymous helpline did not reach the call volume expected, the authors attributed the lower volume to either a lack of knowledge of the service or a fear of stigma, which is well established [22]. There is also a commonly held belief among communities that consider themselves heroes or rescuers that they are unable to stop work to receive help for themselves [23]. In discussions with local law enforcement offices, the authors ascertained that law enforcement officers prefer to discuss traumatic experiences with their peers versus with counselors or mental health professionals. The extant literature also explains privacy concerns held by many first responders [24]. First responder employees seldom engaged with their department’s employee assistance programs for fear of losing their job [25]. Reducing mental health and substance use stigma could encourage first responders and HCWs to seek assistance [26]. The authors also contend that available resources outside one’s employer may facilitate a greater sense of privacy.

### Limitations

Over the course of this project, various limitations were observed and warrant discussion for organizations seeking to support first responders. The advent of COVID-19 suspended the option of in-person instruction. The program adapted to provide the learning modules in an asynchronous format, which forced a curriculum revision to account for the loss of essential interpersonal components of learning. The collection of satisfaction data during synchronous trainings was complicated by participants not being allowed to carry mobile devices in some cases. Additional efforts will be made in future sessions to streamline efforts to provide back-up paper surveys and follow-up to collect data.

Regarding the helpline, we offer two key suggestions. Our first suggestion is to use a peer-based approach. Although the helpline model did employ peer support provided by persons with lived experience with substance use and mental health concerns, the ideal peer-based approach among this group should include first responders helping first responders and HCWs helping HCWs. The second suggestion encourages resource providers to understand this population’s unique privacy requirements. Resource development should reflect a deep understanding of frontline workers’ workplace culture, including prevalent obstacles to their seeking and securing treatment. This paper has included two checklists (Tables S1 and S2), which will assist other organizations to setup these resource frameworks.

## 5. Conclusions

Future research should focus on improving providers’ understanding of this population, particularly care-seeking behavior for mental health and substance use disorders among frontline workers. Law enforcement is particularly challenging to reach, and understanding the needs of this population could improve care delivery. For healthcare and emergency response professionals, attitudes and beliefs about the nature of substance use and mental health disorders affect their professional effectiveness alongside their health and well-being. These populations face unique obstacles to seeking and receiving treatment. The authors’ attempted to address this barrier through a broad educational campaign and helpline to normalize and encourage dialogue regarding mental illness and substance use disorders. The overall goal of this project was to facilitate attitude and behavior change to prevent unnecessary suffering using evidence-based content and support. It was also essential to provide a framework for developing resources to aid frontline workers’ well-being and share these experiences and challenges to assist groups working with this important population.

## Figures and Tables

**Table 1:** Educational campaign for frontline workers, 1 April 2020 until 30 April 2023, *N* = 2151.

Individual-Level Variables	*N* (%)
Total Participants	2151 (100%)
Race	
Black or African American	511 (23.76%)
American Indian or Alaska Native	173 (8.04%)
Asian	546 (25.38%)
White	493 (22.92%)
Native Hawaiian or Pacific Islander	0 (0.00%)
Declined Answer	428 (19.90%)
Ethnicity	
Hispanic/Latino	504 (23.43%)
Non-Hispanic/Latino	1482 (68.90%)
Other/Declined Answer	165 (7.67%)
Sex	
Female	965 (44.86%)
Male	1009 (46.91%)
Non-Binary or Third Gender	2 (0.09%)
Other/Declined Answer	175 (8.14%)
Age	
18–24	169 (7.86%)
25–34	478 (22.22%)
35–44	517 (24.04%)
45–54	554 (25.76%)
55–64	297 (13.81%)
65 or older	115 (5.35%)
Other/Declined Answer	21 (0.98%)
Occupation	
Emergency Medical Services/Paramedic	1031 (47.93%)
Law Enforcement Officer	50 (2.32%)
Firefighter	355 (16.50%)
Other First Responder	667 (31.01%)
Non-First Responder/Declined to Answer	48 (2.23%)

Note: Percentages may not add up to 100 due to rounding.

**Table 2:** List of module topics for frontline workers, as of April 2023.

Substance Use Disorder in First Responders
Coping with Anxiety
Communities with Chronic Conditions
Medications for Opioid Use Disorder
Resilience and Coping with Stress
How to Use Narcan
How to Successfully Address Opioid Overdose
Understanding Suicide
Opioid Crisis in Today’s Youth
Leadership Support: Culturally Competent Care
Alcohol Use Disorder
Managing Your Distress Tolerance

## Data Availability

Data from this study may be obtained by reasonable request by contacting the corresponding author.

## Supplementary Materials

The following are available online at https://www.mdpi.com/article/10.3390/ijerph20206935/s1.
