# Supplementary material for: Helping Frontline Workers in Texas—A Framework for Resource Development
Source: Int J Environ Res Public Health. 2023 Oct 17;20(20):6935. doi: 10.3390/ijerph20206935 (PMC10606751; doi:10.3390/ijerph20206935)
Supplement: Supplementary file 1 [file ijerph-20-06935-s001.zip › Table S1..pdf]

Table S1.

Educational Campaign	Yes	No
Secure learning management system (LMS) platform		
Receive authorization to provide certified continuing education credits		
Identify topics of interest for population		
Research topic and develop training outline		
Create presentation		
Record voice-over		
Create LMS course for each module; populate with presentation		
Create quiz		
Generate and deliver continuing education certificate		
Disseminate online course via social media and through various networks		
Offer free in-person training courses to relevant populations		
Maintain database with relevant information about learners		
