# Supplementary material for: Helping Frontline Workers in Texas—A Framework for Resource Development
Source: Int J Environ Res Public Health. 2023 Oct 17;20(20):6935. doi: 10.3390/ijerph20206935 (PMC10606751; doi:10.3390/ijerph20206935)
Supplement: Supplementary file 1 [file ijerph-20-06935-s001.zip › Table S2..pdf]

Table S2.

Heroes Helpline	Yes	No
Phase I: Conception		
Identify need		
Brainstorm potential solutions		
Select most feasible solution		
Phase II: Development		
Identify potential stakeholders		
Secure funding		
Identify risk factors unique to helpline target population		
Create best practices: protocol, decision tree, script		
Establish relationships and build network of treatment providers		
Create script and training curriculum for peer helpline operators		
Ensure that staff and volunteers are well versed in effective warmline intervention tools and handoff support (SBRIT, motivational interviewing, warm handoff)		
Recruit helpline operators trained in crisis counseling and peer support		
Develop marketing collateral for promotion of service		
Phase III: Implementation		
Set 24/7 call answering schedule for peer operators		
Create contingency support procedure (i.e., call answering service)		
Leverage community partnerships to promote services		
Utilize educational campaign as a resource for marketing		
Disseminate marketing collateral, establish social media presence, create website		
Phase IV: Operational		
Direct emergency callers to 911 or 988		
Listen to caller, recommend navigation services, create a plan for warm handoff		
Follow up with caller within 72 hours		
Record call logs in data management system		
Maintain monthly records of callers		
Record call log in data management system		
